# Microscopic and quantitative characterization of germanium-indium bearing by-product from heavy metal metallurgy

**DOI:** 10.1038/s41598-024-82790-0

**Published:** 2025-01-13

**Authors:** Andrzej Piotrowicz, Piotr Noga, Tomasz Skrzekut, Gabriela A. Kozub-Budzyń, Maciej Wędrychowicz, Dominika Skarupska

**Affiliations:** 1 Faculty of Mechanical Engineering, Institute of Materials and Biomedical Engineering, University of Zielona Gora, prof. Z. Szafrana 4 Street, Zielona Gora, 65-516 Poland; 2 Faculty of Non-Ferrous Metals, AGH University of Krakow, A. Mickiewicza Av. 30, Cracow, 30-059 Poland; 3 Faculty of Geology, Geophysics and Environmental Protection, AGH University of Krakow, A. Mickiewicza Av. 30, Cracow, 30-059 Poland

**Keywords:** Structural materials, Metals and alloys

## Abstract

This article presents the results of study on the material characterization of germanium-indium drosses (Ge-In-D). Ge-In-D are a by-product of obtaining zinc and lead, which are currently not processed yet. Due to the exceptionally high concentrations of germanium and indium in them, as well as the commercial value of these elements, it became important to properly identify Ge-In-D, which was the aim of this work. Ge-In-D were characterized quantitatively and microscopic analyzes were also performed. The chemical composition of Ge-In-D was determined as follows (percentage by mass): 27.195% Sn; 20.737% Pb; 15.764% Cu; 9.782% As; 9.274% Ge; 7.875% In; 3.872% Fe; 2.617% Ag; S, Ni, Zn, Ga, Se, Cd, Sb as the rest. The combination of granulometry and chemical analyzes shows that germanium and indium tend to accumulate in fine fractions.

## Introduction

Materials containing Ge and/or In are of all types: from minerals and concentrates, by-products of large-scale metallurgy, to municipal waste and end-of-life products. These materials differ primarily in their chemical composition and origin. The contents of Ge and In in them can reach up to 10 and 6 wt%, respectively, although they are often below 1 wt%. These products are the result of various hydro- and pyrometallurgical processes for obtaining other metals such as zinc, lead and copper^1–4^. Table 1 shows intermediates with known chemical compositions, containing Ge and In. The germanium-indium drosses (Ge-In-D) discussed in this work are a by-products of zinc and lead metallurgy, hence the technological processes related to these metals will be presented later.

**Table 1:** Chemical compositions of germanium and indium by-products from heavy metal metallurgy.

Type	Source / ref.		In	Ge	Sn	Pb	Cu	Zn	Sb	As	Bi	Ag	Tl	Fe	Al
			% mass.												
indium-germanium concentrates	^5^		3,17 − 4,05	0,25 − 0,30	–	7,04–8,10	–	20,03–22,34	–	–	–	175–202 [ppm]	–	–	–
indium drosses	*HC Miasteczko Śląskie*,* Poland* / PbSnIn	^1,2^	0.23–0.50	0.012	2.62–3.91	93.3-93.73	0.0029	1.45–6.23	0.076–0.17	0.0024-0.02	0.03–0.20	–	0.031	–	–
retort drosses	*HC Miasteczko Śląskie*,* Poland* / PbSnCuGeIn	^1,2^	1.74	6.90	35.9	19.0	21.4	8.63	1.06	1.55	0.061	1.55	–	–	–
	*MHK Zletovo-Veles*, Macedonia/ Pb-Zn wastes	^3^	2	–	11	72	2.5	10	1.5	1	–	–	–	0.15	–
germanium-iron solid residues	vacuum distillation of solid residues from zinc rectification	^4^	-	3.3–10.6	36.5–37.2	14.7–18.5	10.2–22.5	17.4–1.92	-	–	–	–	–	–	3.58–3.6
liquation drosses	HM Legnica	^6^	50 [ppm]	–	–	65	12	–	–	–	–	–	–	–	–
	HC Miasteczko Śląskie	^6^	50–600 [ppm]	–	–	25–60	15–35	–	–	–	–	–	–	–	–
	MHK Zletovo-Veles	^7^	300–1200 [ppm]	–	–	60–65	20–30	1,0–2,5	1,5 − 2,5	–	–	–	–	–	–
sulphide drosses	HC Miasteczko Śląskie	^6^	50 [ppm]	–	–	50–80	5–15	–	–	–	–	–	–	–	–
zinc drosses	^8^		3,23	–	12,63	62,2	–	1,02	–	–	–	–	–	–	–
blast furnace slime	ZHiS Maanshan	^9^	0,0169	–	–	1,00	–	2,17	–	–	–	–	–	36,57	5,54

Pyro- or hydrometallurgical methods are used to produce zinc from primary or secondary sources. Technologies for zinc metallurgical production are divided into: - horizontal batch process, - in a vertical retort, - Imperial Smelting Process (*ISP*,* Ltd*), - in an electric furnace^10^. A typical *ISP* consists of several stages: (1) Preparation of the charge (briquette/sinter), (2) Smelting of Zn and Pb in a shaft furnace (smelting furnace, ISF), (3) Rectification of Zn and Cd.

Zn-Pb concentrates are mixed with residues from previous process and fine sinter fractions, with secondary material and other technological materials. The mixture is fed to a sintering machine with upper/lower draft and ignited. During sintering, the material is transported through a series of air boxes. As a result of sintering, sulfides are oxidized and mainly SO_2_ is released, and the heat generated is sufficient to melt and sinter the material, resulting in a sinter – Zn and Pb concentrate devoid of most of the sulfur, at the same time porous enough to be permeable to gases, and mechanically durable. SO_2_ is sucked in by the suction nozzle and sent for disposal into H_2_SO_4_^11,12^.

Sintering takes place in an shaft furnace. Hot sinter (~ 45% ZnO, ~ 20% PbO) together with hot metallurgical coke and fluxes are loaded into the furnace through a bell system. The oxidizer, i.e. air or air enriched with oxygen, is blown through nozzles into the furnace. The coke oxidation reaction occurs, which, under appropriate conditions, favors the Boudouard reaction, which is mostly responsible for the reduction reactions of metal oxides in the sinter. High temperature (up to 1300 °C) and reducing conditions cause the melting of metals, of which zinc, due to its high vapor pressure, distills along with the furnace exhaust gases. The furnace gases pass through a spray condenser, in which a downpour of liquid lead (spraying) cools the gases to approximately 450–500 °C. The zinc condenses in the lead. This treatment prevents Zn reoxidation (cooling of Zn vapor occurs without the access of the oxidant). The resulting Zn-Pb alloy separates as a result of its free cooling: Zn, being lighter, forms the upper layer in the liquefaction bath, and the heavier lead forms the lower one. Some of Pb is returned to the condenser^11,12^.

Raw zinc, due to a certain amount of impurities, especially Cd, Cu and Pb, should be refined in rectification columns. The columns consist of refractory shelves through which metal vapors flow. There is an overflow hole at the bottom of each box. Adjacent boxes are positioned relative to each other so that the liquid metal or its vapors must flow from one box to the other in a zigzag manner. The lower parts of the columns are heated, and the upper parts are not - rectification takes place in the dephlegmator. Low-boiling components are rectified, while those with higher temperatures fall to the bottom of the column. In the zinc column, the rectification of Zn takes place, which evaporates together with Cd, and in the cadmium column, the separation between Zn and Cd takes place. The distillate from the first column is directly directed to the second column. The metal from the bottom of the second column is high-grade zinc (*SHG*)^12–15^. During the production of primary zinc in the New Jersey process, various types of by-products are produced, which may include Ge and In (Table 1)^1,4,16^.

The goal of this article was to characterize, in terms of material, germanium-indium drosses originating from heavy metals metallurgy. Although the goal may sound prosaic, the literature review shows that there is still no reliable and complete material characterization of Ge-In-D. The literature data contradict each other with the results of the chemical composition of the Ge-In-D, which may be due to several reasons: - the input from which the Ge-In-D are made is variable (oxide or sulfide charge, which determine the composition of the concentrate), - technological parameters vary in different plants, - various scientific methods may give different results. Almost always, the characterization of Ge-In-D are limited only to the analysis of the quantitative chemical composition, which ignores the important issue of microscopic analysis. Due to the above, a number of studies were carried out to characterize Ge-In-D.

## Methods

The research material are germanium-indium drosses derived from the zinc distillation process as a solid residue. This material was tested using several research methods: - granulometric analysis; - microscopic analyzes (optical and electron), - analyzes of quantitative chemical composition.

Due to the nature of the research material, which is characterized by different grain sizes, and the insinuation that some fractions may be richer in Ge and/or In, a sieve analysis of the research material was performed. Sieve analysis was performed based on sieving according to the polish standard PN-90/H-04933, with the difference that the sieving was performed manually (not automated)^17^. Sieve analysis was performed for raw and ground research material. The ground material was prepared by grinding the raw material in a ring-roll mill (*TEST-LAB-09*,* „Eko-Lab”*) for 4 min.

Samples for sieving (dry sample, drying time 24 h, 110 °C, electric dryer *SLN 115 STD*, *Pol-Eko-Aparatura Sp.J.*) were sieved on a set of sieves (*VEB Metallverbei Neustadt/Orla*,* Kombinat NAGEMA*) in the sieve size range from 2.5 to 0.040 mm. Weighing samples for screening in the amount of 100 g ± 0.1 g and the fractions after screening were weighed with an accuracy of 0.1 g (*WPS-360/C*,* Rad-Wag*). Sieving time was 20 min. After sieving, each of the separated fractions was weighed. The sieving loss was added during recording to the mass of the undersize fraction collected in the bottom^17^. Sieving for each material was performed nine times using the same tools and under the same laboratory conditions. In subsequent studies, the separated fractions constituted research material for determining their chemical composition, as well as the shape of grains, etc.

Microscopic investigation was performed. For this purpose, metallurgical specimens of materials and their fractions were performed (*EpoFix resin*,* EpoFix hardener*,* Struers*). Then, the sections were ground *(# 4000*,* MR54*,* Struers*) and a conductive layer of carbon was applied to them using various sputtering machines (*208 carbon*,* Cressington Carbon Coater or Q150TE*,* QUORUM*).

The specimens were used for microscopic and morphological analyses: i.e. optical microscopy (*EPIPHOT 200* metallurgical microscope, *Nikon*), scanning electron microscopy with an energy dispersive spectroscopy system (*SEM-EDS*) and element mapping (field emission gap, *SU-70*,* Hitachi*), and also an electron microprobe equipped with optical microscopes for reflected and transmitted light (*JEOL Super Probe 8230*). These analyzes allowed not only the assessment and determination of the morphology, but also the elemental composition of the materials.

Fractions of the material were additionally examined in terms of chemical composition with an electron microprobe JEOL SuperProbe JXA-8230 (EPMA) at the Laboratory of Critical Elements, AGH University of Science and Technology, Kraków equipped with X-ray wavelength spectrometers (*WDS*) and an X-ray energy dispersive spectrometer (*EDS*). The EPMA was operated in the wavelength-dispersion mode, at the accelerating voltage of 20 kV and probe current of 30 nA for metallic phases and 15 kV and 20 nA for oxides. Focused beam with 1 μm diameter was used for metallic phases, 3 μm for oxides, counting time of 20 s on peak and 10 s on both (+) and (–) backgrounds were applied. The following standards, lines and crystals were used for metallic phases: pyrite (FeKα, LIF), sphalerite (SKα, PETJ; ZnKα, LIF), GeS (GeKα, LIF), AgMet (AgLα, PETH), chalcopyrite (CuKα, LIFH), GaAs (AsLα, TAPH; GaLα TAPH), In_2_Se_3_ (InLα, PETH), CdS (CdLα, PETH), SnS (SnLα, PETL), galena (PbMα, PETL), Sb_2_Se_3_ (SbLα, PETL; SeLα, TAPH) and NiMet (NiKα, LIFL). Whereas the following standards, lines and crystals were used for oxides: albite (SiKα, TAP), tugtupite (ClKα, PETJ), sanidyne (KKα, PETJ), cassiterite (SnLα, PETJ), NiO (NiKα, LIF), cuprite (CuKα, LIFH), Sb_2_Se_3_ (SbLα, PETH), willemite (ZnKα, LIFH), crocoite (PbMα, PETH), fayalite (FeKα, LIFL), anhydrite (SKα, PETL), In_2_Se_3_ (InLα, PETL), AgMet (AgLα, PETL), GaAs (AsLα, TAPH; GaLα TAPH), GeS (GeLα, TAPH). Data were corrected using ZAF procedure. Thanks to this, it was possible to analyze the distribution of elements in the micro-area, as well as to determine the content of metallic and oxide phases.

Fractions were also examined using a scanning electron microscope with an energy dispersive spectroscopy system (*SEM-EDS*) (*SU-70*,* Hitachi*), and point, linear and area analysis of the chemical composition.

### Results and analyses

**Fig. 1:**
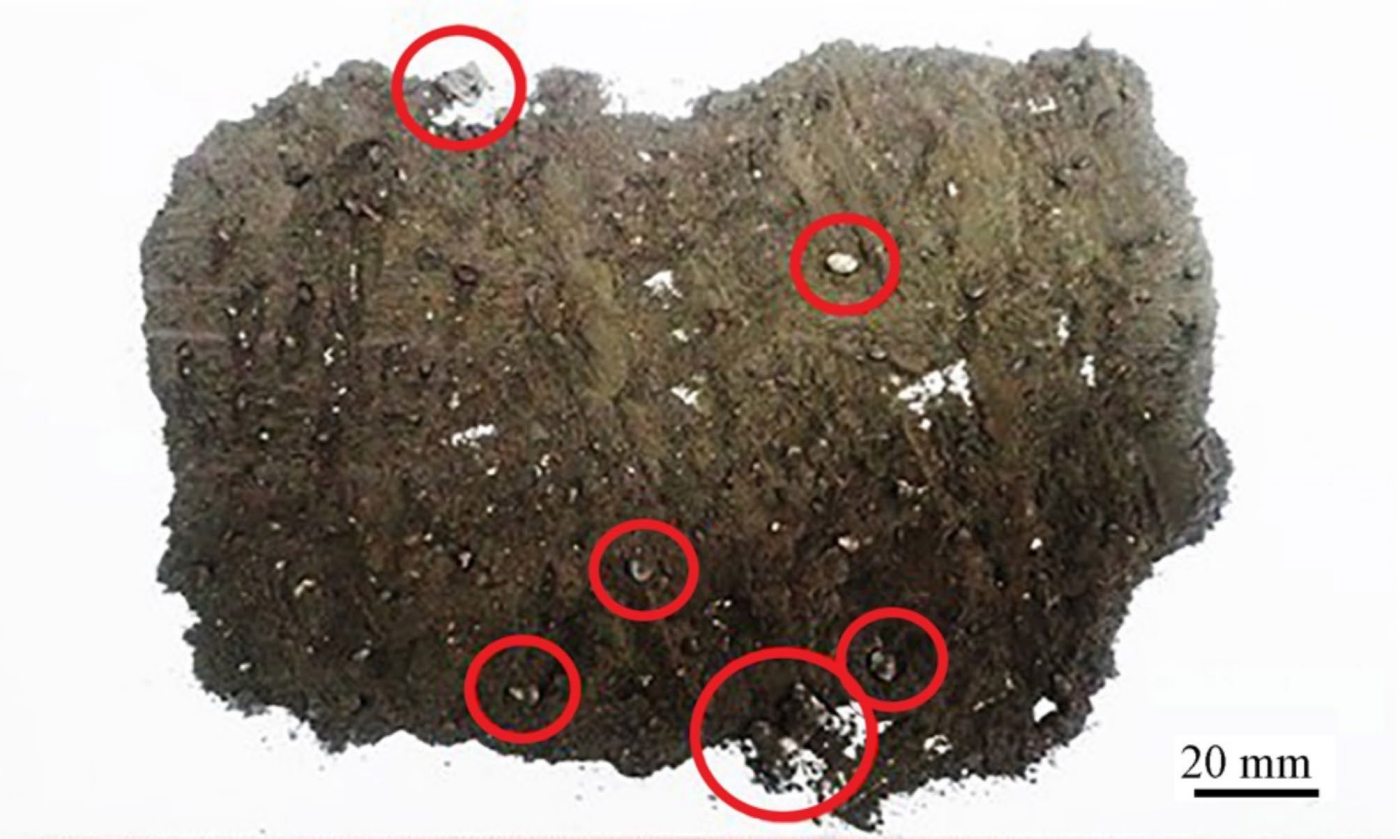
Germanium-indium drosses; fractions of metallic inclusions and drops marked with circles.

The visual apparition of Ge-In-D is shown in Fig. 1. It can be said that Ge-In-D are a heterogeneous mixture of metallic particles (inclusions, drops) and gray-brown fine, dusty powder. Occasionally, white pieces of ceramics could be found, which were the lining of the furnace.

**Fig. 2:**
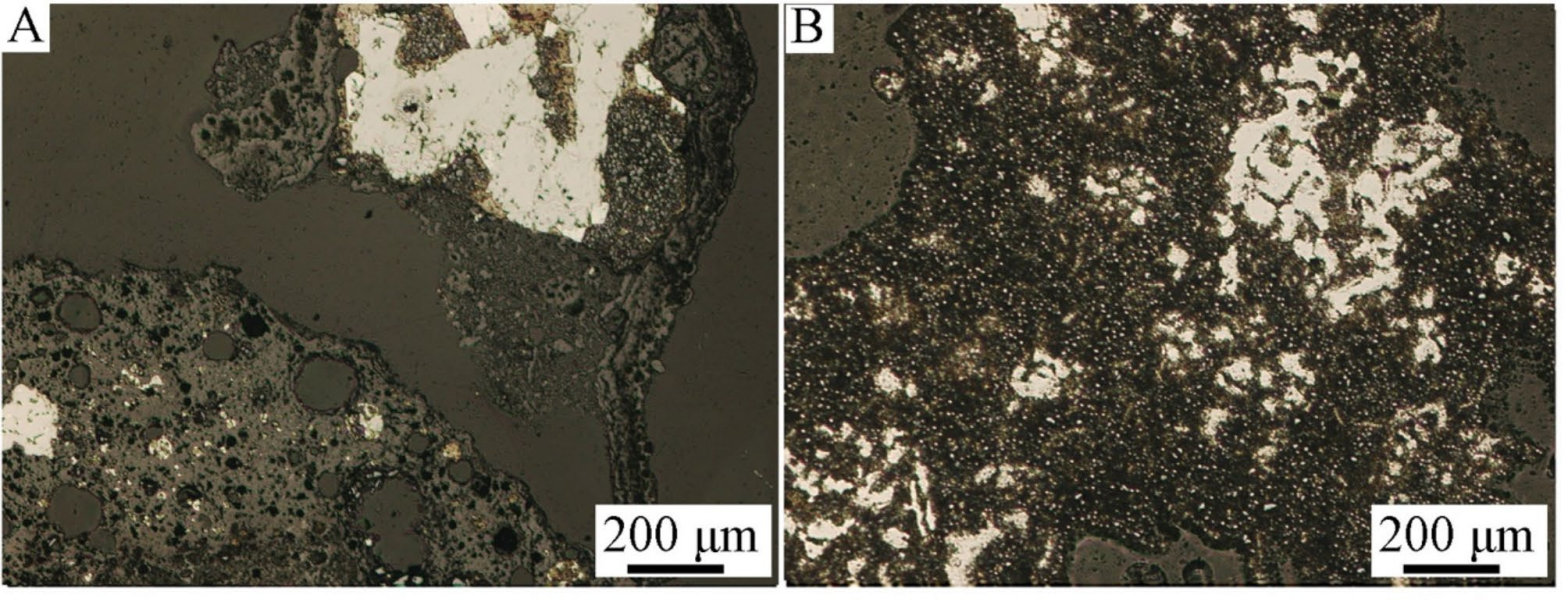
Optical microscopy images of grain class 1.0/2.0 mm, 100x magnification.

**Fig. 3:**
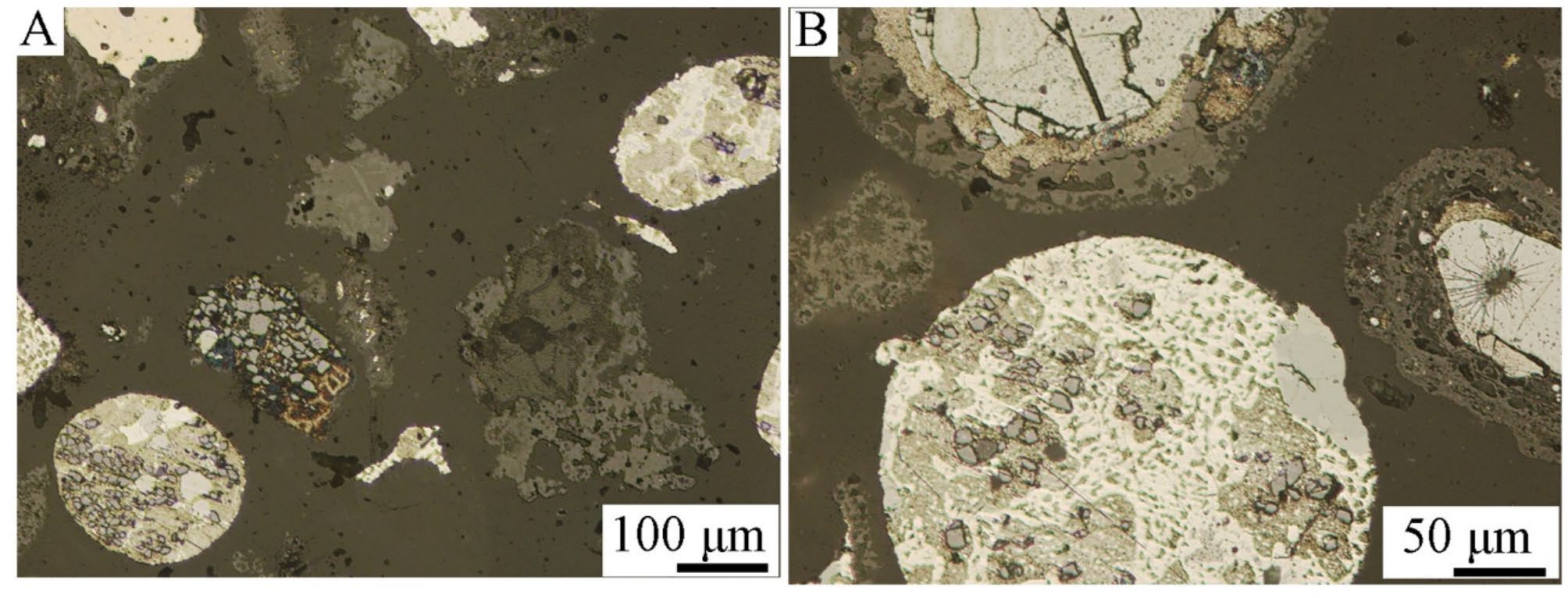
Optical microscopy images of grain class 0.160/0.20 mm, (**A**): 10x, (**B**): 20x magnification.

**Fig. 4:**
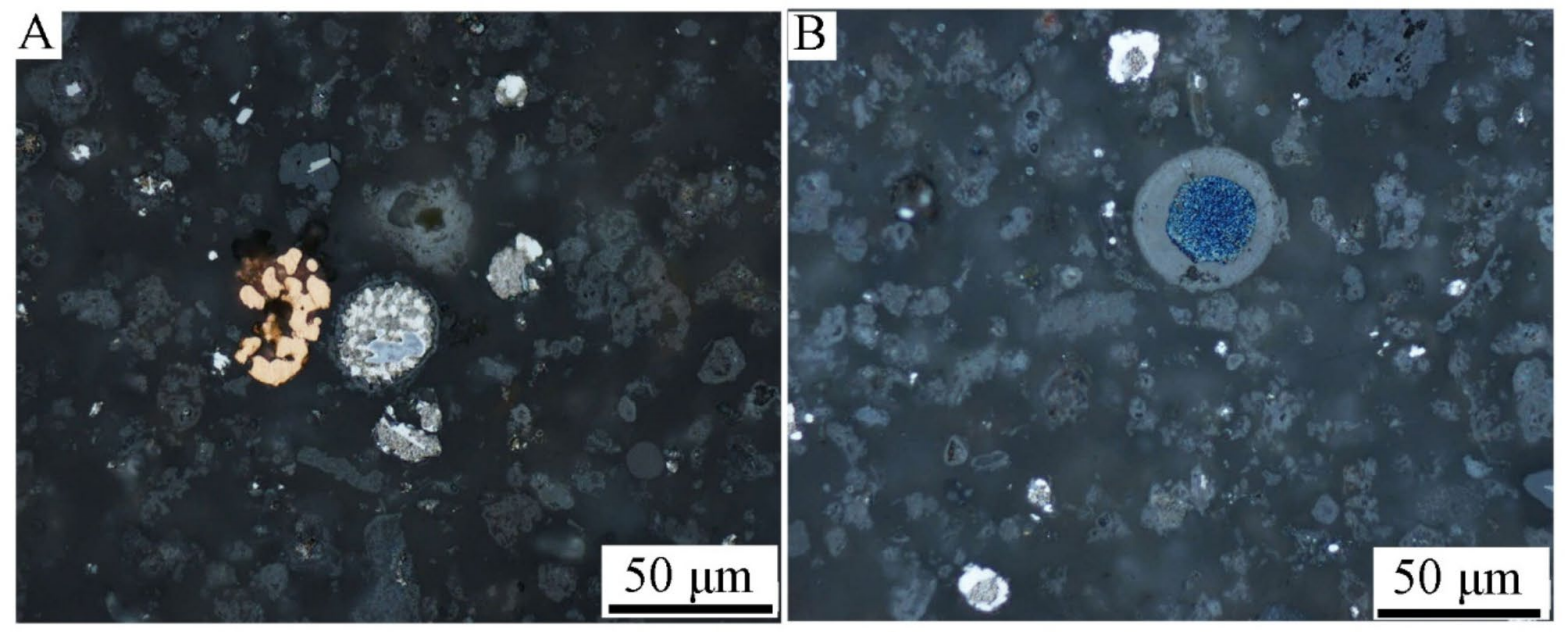
Optical microscopy images of grain class < 0.040 mm, 50x magnification.

Figures 2, 3 and 4 show optical microscope photos of various grain classes of Ge-In-D. The material is heterogeneous in particle shape and morphology, and possibly composition, as evidenced by the different colors. In Fig. 4 can be clearly seen metallic copper (A, orange piece in the middle of the photo) and a blue-colored phase (B), probably Cu, Zn or Cd alloy. The structure of round grains is characteristic, which consist of a clear “shell” (in the photos as a dark outer shell of the grains) and a light “core” (Figs. 3B and 4: grains with a light and blue center). Segregation within grains may imply the chemical composition of individual parts and, consequently, different chemical composition results depending on the research method. Hence, there was a need to examine the chemical compositions of individual parts of the grains, especially the “shell” and “core” of the grains.

**Fig. 5:**
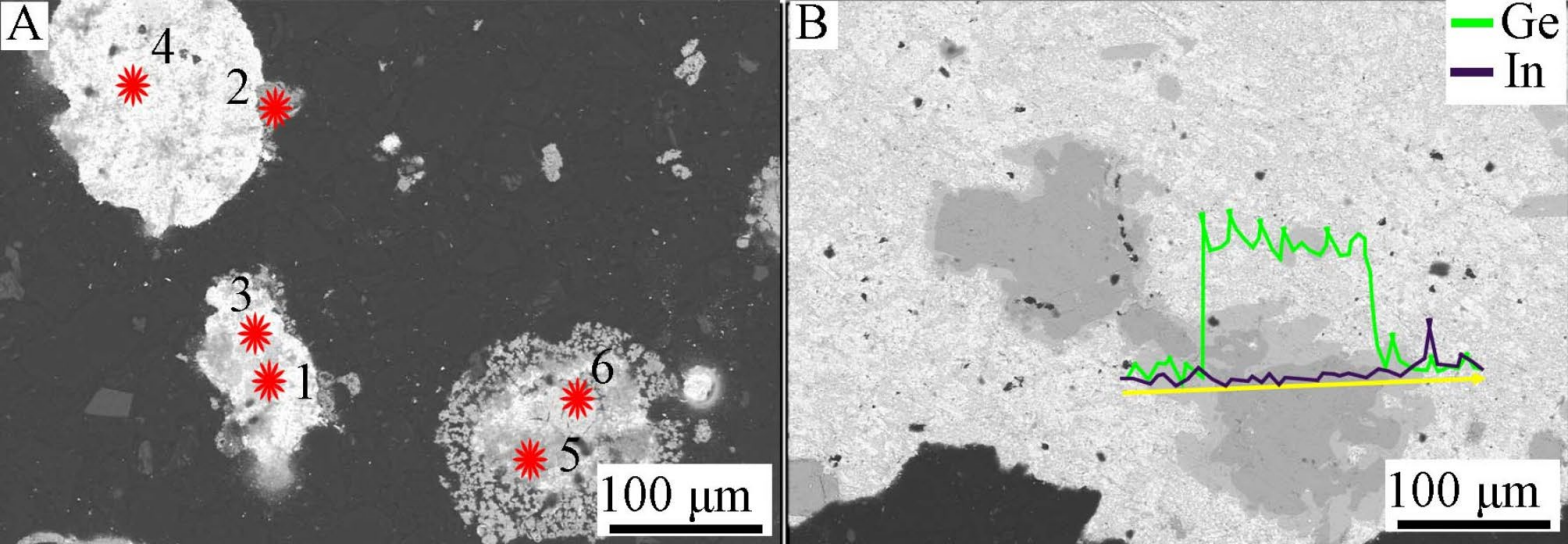
*BSE* image of grain class 1.0/2.0 mm: (**A**)—points of point composition analysis, (**B**)—line of linear composition analysis.

**Fig. 6:**
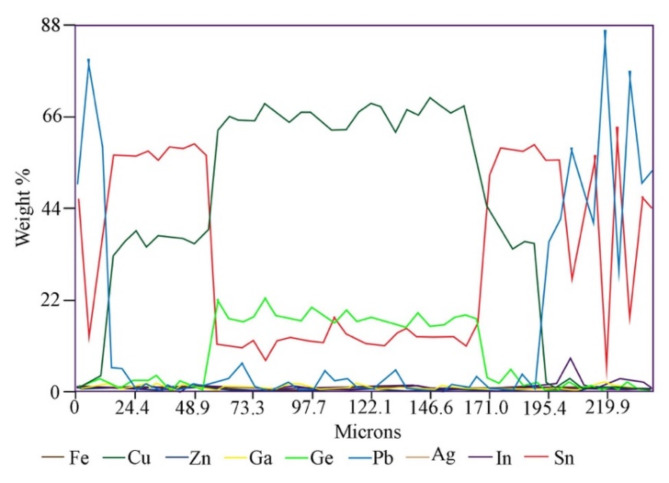
Linear composition analysis of grain class 1.0/2.0 mm using *WDS-EDS* method (as in Fig. 5B).

Point and linear analyzes of the chemical composition of grain classes show that they are not chemically homogeneous. Below is an example with a grain class of 1.0/2.0 mm (Fig. 5; Table 2). The “core” of the grains consists mainly of either a copper-tin alloy in the proportions 38.23/56.67 (wt%), lead with small admixtures of other metals (up to 82–92 wt% Pb) or from a Sn-Pb alloy in the proportions 15.75/82.37 (wt%). However, the “shell” consists of Zn − 39.12 wt%, Sn − 27.40 wt%, but most importantly, also in large amounts of Ge and In, 17.48 and 3.46 wt%, respectively. A linear analysis of the chemical composition along the grain (Fig. 6) shows that Ge rather accumulates in the “core” (up to 22 wt%), while indium is located primarily in the “shell”. The main component of the “core” is Cu (on average 66 wt%), and the “shell” consists of tin (up to 66% by weight) and Pb (up to 88 wt%), as well as some Cu.

**Table 2:** Point composition analysis of grain class 1.0/2.0 mm using *WDS-EDS* method (as in Fig. 5A).

No.	Fe	Cu	Zn	Ga	Ge	Ag	In	Sn	Pb
	% mass.								
1	0.05	56.67	0.00	0.00	0.74	0.00	0.39	38.23	3.92
2	0.80	0.85	39.12	1.71	17.48	0.16	3.46	27.40	9.01
3	0.19	0.73	0.22	0.00	0.44	0.06	0.45	5.00	92.75
4	0.16	0.10	0.52	0.00	0.10	0.12	0.88	15.75	82.37
5	0.50	0.27	1.69	0.00	2.86	0.33	3.80	30.82	59.72
6	0.00	1.98	0.00	0.00	0.32	0.00	47.44	10.85	39.41

**Fig. 7:**
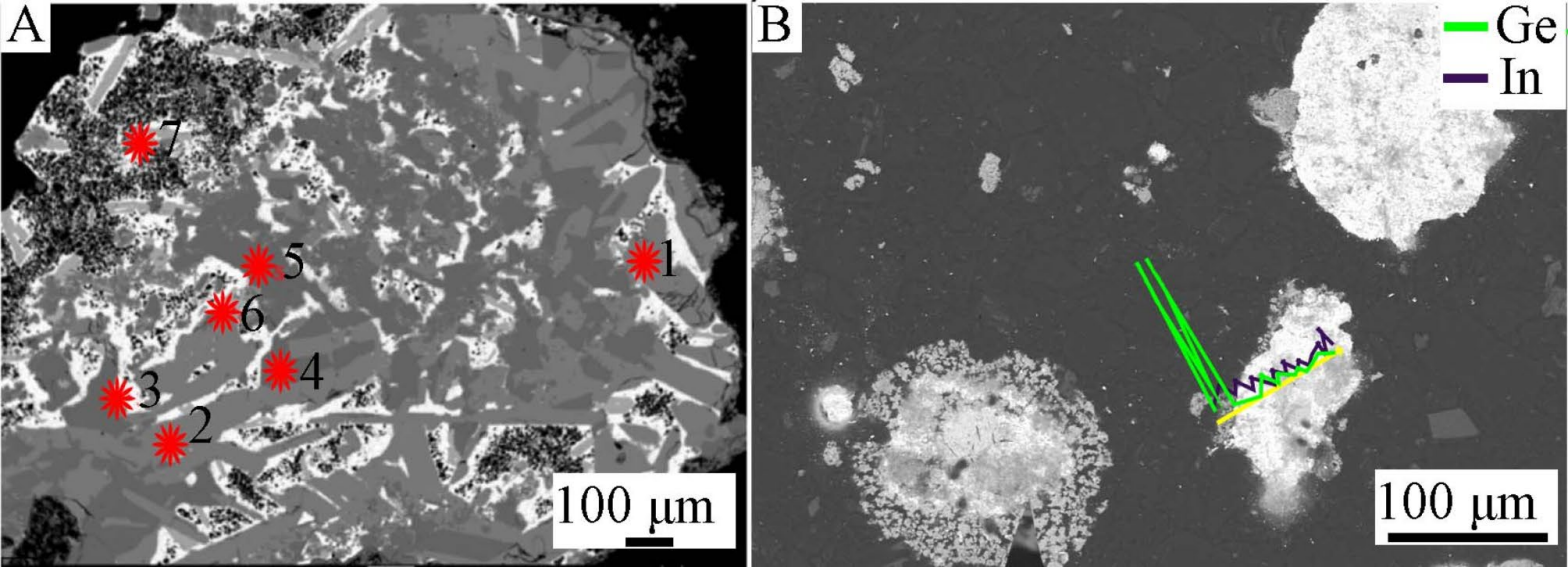
*BSE* image of grain class 0.160/0.20 mm: (**A**)—points of point composition analysis, (**B**)—line of linear composition analysis.

**Fig. 8:**
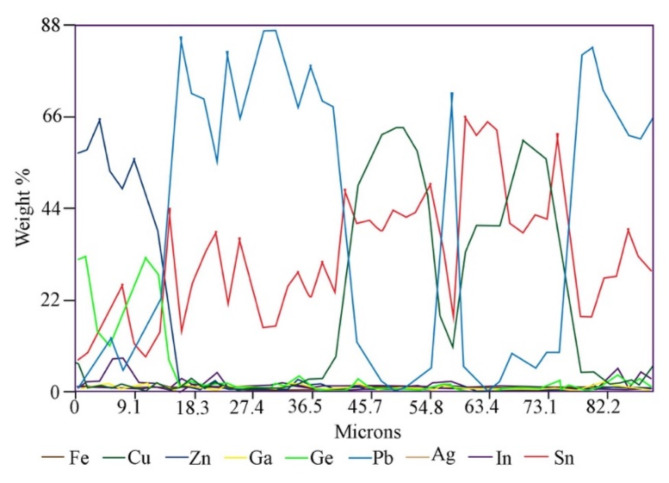
Linear composition analysis of grain class 0.160/0.20 mm using *WDS-EDS* method (Fig. 7B).

The results of point and linear analyzes for another grain class, 0.160/0.20 mm (Figs. 7 and 8; Table 3) show that the color of the area in the photos depends on the chemical composition of the examined area. The lightest areas (point 3, Fig. 7) are Pb (93.215 wt%) with a small Sn content (3.462 wt%). The slightly darker areas (points 6 and 7) consist of Sn (approx. 56 wt%), As (approx. 29 wt%) and Pb (approx. 11 wt%) and do not contain Ge. The gray areas (points 1 and 2) are a Cu-Sn alloy with an average composition of 64 wt%/35.5 wt%, Cu/Sn, respectively. Finally, the darkest areas (points 4 and 5) are an alloy with an average composition of 70–72 wt%. Cu and 27.5–30 wt% As. The investigated grain turned out to be low in Ge and In. However, the linear analysis of the composition (Fig. 8) shows that Ge and In are located primarily in the “shell” with concentrations of approximately 30 and 6 wt%, respectively; this also applies to Zn, which contained up to 63 wt%. The “core” includes Cu, Sn and Pb in various proportions.

**Table 3:** Point composition analysis of grain class 0.160/0.20 mm using *WDS-EDS* method (Fig. 7A).

No.	Fe	Cu	Zn	Ge	As	Ag	In	Sn	Pb
	% mass.								
1	0.011	64.272	0.046	0.200	0.390	0.050	0.225	35.593	0.159
2	0.019	64.111	0.007	0.464	0.302	0.085	0.243	35.473	0.025
3	0.009	0.428	0.000	0.124	0.000	0.000	0.000	3.462	93.215
4	0.003	71.920	0.058	0.000	27.568	0.045	0.000	0.561	0.045
5	0.059	70.172	0.084	0.082	29.907	0.032	0.017	0.517	0.023
6	0.020	0.089	0.017	0.000	29.225	0.000	0.490	56.772	10.899
7	0.003	0.000	0.032	0.000	29.575	0.000	0.502	56.575	11.684

**Fig. 9:**
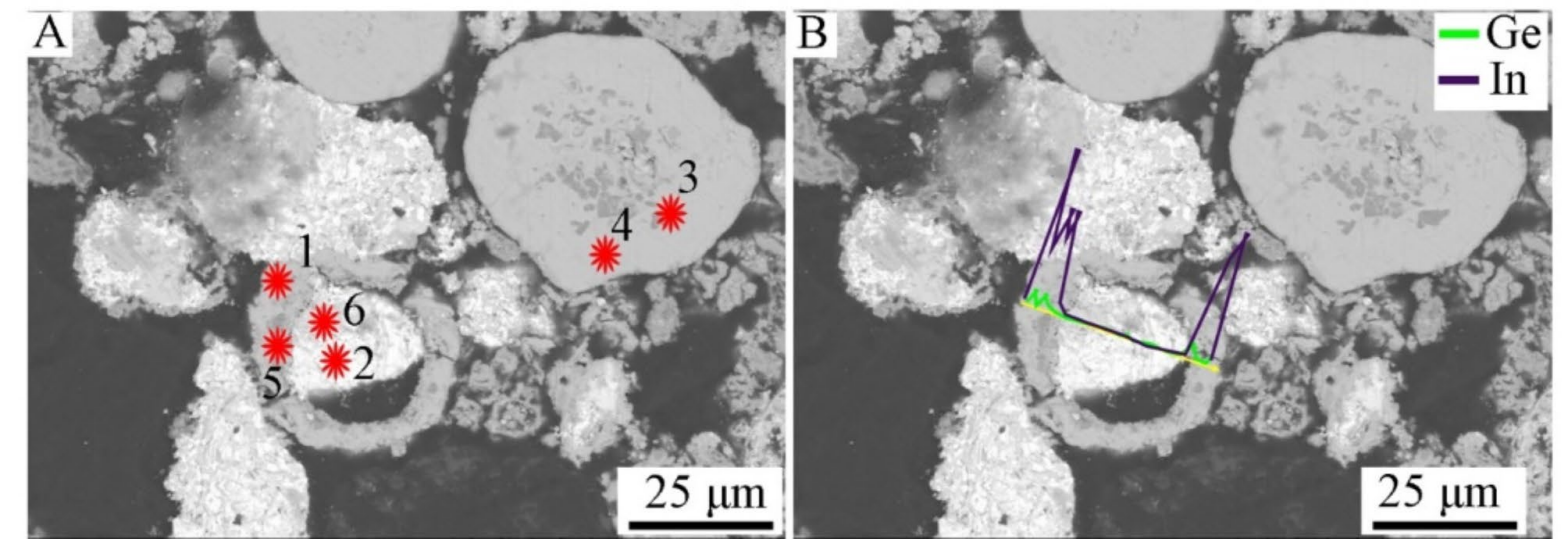
*BSE* image of grain class < 0.040 mm: (**A**)—points of point composition analysis, (**B**)—line of linear composition analysis.

**Fig. 10:**
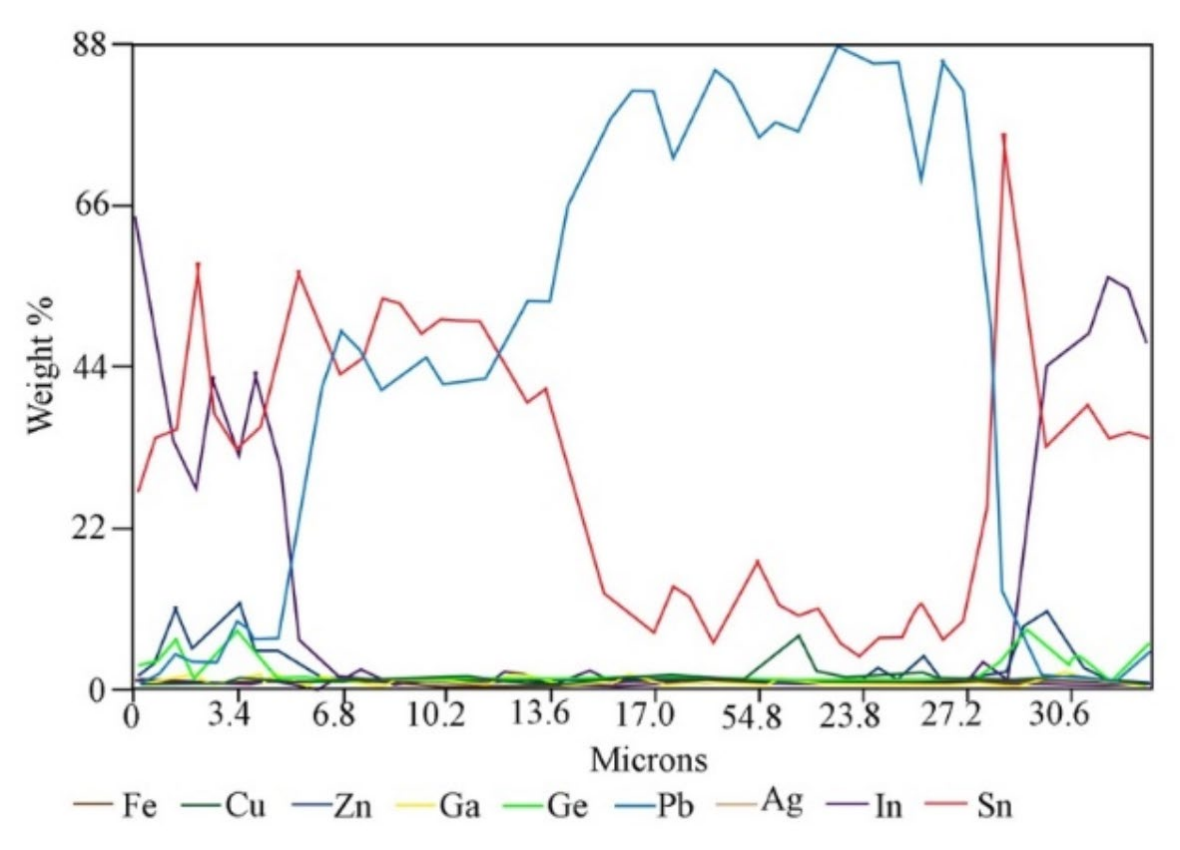
Linear composition analysis of grain class < 0.040 mm using *WDS-EDS* method (Fig. 9B).

Point analysis of the grain class < 0.040 mm (Fig. 9) showed that the dark areas are exceptionally rich in Ge and In, up to 30 and 38.5 wt%, respectively (Table 4), and the darkest ones are rich in Ge and Zn (approx. 31 and 51 wt%, respectively). This applies not only to the “shell”, but also to whole grains, the *BSE* image of which is gray or dark gray. However, linear analysis along the grain (Fig. 10) confirms that the “shell” is exceptionally rich in In, up to 69 wt%. and that it also contains Ge and Zn (a few wt%) and a lot of Sn. The “core” consists mainly of Sn and Pb. Hence, it can be assumed that the most In should be found in the smallest fraction of Ge-In-D.

**Table 4:** Point composition analysis of grain class < 0.040 mm using *WDS-EDS* method (Fig. 9A).

No.	Fe	Cu	Zn	Ga	Ge	In	Sn	Pb
	% mass.							
1	0.25	0.06	2.96	0.78	2.04	28.15	59.56	3.75
2	0.06	0.43	0.32	0.06	0.68	0.89	3.93	93.64
3	2.59	0.05	50.84	0.08	30.93	6.84	7.62	1.05
4	0.00	0.42	0.50	0.33	1.97	38.50	55.71	2.05
5	2.19	0.16	3.44	0.00	4.23	10.83	62.64	16.51
6	0.02	0.09	0.55	0.00	0.41	0.84	54.21	43.87

Table 5 presents the chemical compositions of grain classes determined by the *WDS-EDS* method. Fe in all grain classes was on average from approx. 2 to 6 wt%. The Cu content ranges from 7 to over 20 wt%, of which it was the lowest for the grain class 0.040/0.056 mm. The concentrations of Ga, similarly to Ge and In, were the highest in the smallest grain classes, i.e. from 0.056 to < 0.040 mm, and were respectively: approx. 0.035; approx. 15 and approx. 16 wt%. Apart from the 0.071/0.100 mm grain class, the Ge content in the remaining grain classes was in the range of 6–12 wt%; in the case of In, it was in the range of 5–6 wt%. As with a content of 12–16 wt%. it was most abundant in thick fractions and in the 0.071/0.100 mm fraction. The Ag content was the highest in coarse grains, from > 2.5 to < 0.50 mm, and amounted to 4–6 wt%, while in the remaining grain classes it was only a maximum of 1.5 wt%. Sn content in all grain classes was in the range of 20–35 wt%. It turned out that the most lead, 30–35 wt%, is found in grain classes 0.160/0.315; 0.100/0.160; 0.071/0.100 and 0.056/0.071 mm. It quite often happened that the analyzed points consisted of either only one element or several, as evidenced by the recorded maximum concentrations of the analyzed elements. They were probably pure metals or their low-melting alloys. The average composition of Ge-In-D determined by the *WDS-EDS* method is as follows (wt%): 3.872 Fe; 15.764 Cu; 9.274 Ge; 9.782 As; 2.617 Ag; 7.875 In; 27.195 Sn and 20.737 Pb.

**Table 5:** Chemical composition germanium-indium drosses determined by WDS-EDS method.

Grain class, mm		S	Fe	Ni	Cu	Zn	Ga	Ge	As	Se	Ag	Cd	In	Sn	Sb	Pb
		% mass.														
2.0/2.5	Maximum concentration	0.096	50.815	4.994	73.025	1.960	0.259	77.563	54.894	0.528	74.190	0.377	60.837	95.922	4.515	95.584
	Average concentration (63 pts)	0.010	6.078	0.504	19.477	0.096	0.017	7.101	16.275	0.027	5.904	0.056	5.697	22.04	0.466	14.639
	Standard deviation	0.017	14.790	1.053	30.182	0.338	0.055	20.322	20.039	0.085	20.114	0.110	17.084	27.850	0.804	32.713
1.0/2.0	Maximum concentration	0.045	43.177	3.218	73.283	0.093	0.285	74.005	54.453	0.293	73.793	0.365	60.782	96.284	2.391	95.118
	Average concentration (56 pts)	0.007	4.436	0.304	23.159	0.024	0.016	5.803	15.960	0.014	5.286	0.035	5.401	27.655	0.448	10.141
	Standard deviation	0.010	12.682	0.708	31.646	0.029	0.058	16.141	18.550	0.046	19.104	0.085	16.682	29.909	0.629	26.854
0.5/1.0	Maximum concentration	0.064	43.175	3.902	72.311	0.104	0.258	79.520	53.016	0.537	73.191	0.383	61.265	95.393	2.797	97.515
	Average concentration (57 pts)	0.008	5.804	0.399	17.672	0.021	0.018	6.948	13.356	0.034	3.852	0.059	5.400	26.367	0.433	17.815
	Standard deviation	0.012	14.344	0.905	29.023	0.028	0.062	17.657	19.647	0.120	16.327	0.120	16.946	30.901	0.728	35.848
0.315/0.50	Maximum concentration	0.025	42.094	7.095	73.570	0.119	0.439	78.415	52.697	0.603	75.699	0.389	62.650	95.682	2.670	97.455
	Average concentration (64 pts)	0.005	4.661	0.526	21.661	0.027	0.031	11.679	12.835	0.039	4.731	0.044	6.722	24.224	0.319	11.357
	Standard deviation	0.006	12.868	1.195	30.835	0.033	0.094	21.834	18.697	0.108	18.351	0.097	18.679	28.926	0.584	29.177
0.160/0.315	Maximum concentration	0.039	41.538	3.179	71.463	0.090	0.207	44.851	61.065	0.334	41.66	0.385	34.579	95.248	2.559	98.090
	Average concentration (57 pts)	0.004	2.727	0.201	13.445	0.046	0.008	6.748	5.640	0.017	1.225	0.124	4.899	29.305	0.164	33.199
	Standard deviation	0.010	12.850	0.584	28.809	0.029	0.046	21.343	20.147	0.067	9.605	0.124	10.026	30.612	0.658	37.097
0.100/0.160	Maximum concentration	0.037	41.079	3.065	71.312	0.104	0.200	41.803	61.702	0.322	38.570	0.386	32.052	95.182	2.480	98.301
	Average concentration (43 pts)	0.005	3.330	0.240	16.236	0.055	0.009	8.235	6.766	0.021	1.346	0.038	1.691	24.287	0.197	35.039
	Standard deviation	0.009	12.753	0.552	28.644	0.017	0.045	20.650	20.221	0.066	8.704	0.126	9.355	30.754	0.653	37.682
0.071/0.100	Maximum concentration	0.036	40.901	3.021	71.254	0.075	0.195	40.624	61.953	0.318	37.374	0.386	31.074	95.157	2.450	98.383
	Average concentration (30 pts)	0.004	2.696	0.193	13.105	0.044	0.008	6.660	5.442	0.017	1.038	0.031	1.325	29.776	0.159	34.566
	Standard deviation	0.009	12.715	0.540	28.580	0.012	0.044	20.382	20.249	0.065	8.356	0.127	9.095	30.809	0.65	37.908
0.056/0.071	Maximum concentration	0.047	41.739	0.000	69.487	0.392	0.000	3.135	71.426	0.046	1.069	0.383	0.646	94.878	2.698	98.656
	Average concentration (23 pts)	0.010	4.575	0.000	17.298	0.094	0.000	0.405	13.978	0.002	0.107	0.097	0.154	29.986	0.407	31.201
	standard deviation	0.012	12.369	0.000	27.149	0.082	0.000	0.848	21.654	0.010	0.234	0.147	0.191	31.342	0.706	43.942
0.040/0.056	Maximum concentration	0.035	40.771	2.988	71.211	0.529	0.195	79.759	71.137	0.314	1.497	0.387	30.357	95.138	2.428	98.443
	Average concentration (16 pts)	0.005	3.131	0.222	7.599	0.153	0.044	15.454	6.296	0.020	1.168	0.035	15.074	34.804	0.184	13.508
	Standard deviation	0.009	12.688	0.530	28.534	0.008	0.044	10.186	20.270	0.065	0.101	0.127	0.905	30.850	0.649	38.074
0.00/0.040	maximum concentration	0.035	40.723	2.976	71.196	0.45	0.195	79.444	71.204	0.313	1.178	0.387	30.096	95.131	2.419	98.465
	Average concentration (10 pts)	0.004	2.342	0.166	11.369	0.114	0.033	14.447	4.702	0.015	0.861	0.026	16.747	26.125	0.138	20.305
	Standard deviation	0.009	12.678	0.527	28.517	0.007	0.044	10.114	20.278	0.065	0.001	0.127	0.836	30.864	0.648	38.134
Weighted arithmetic mean according to the share of grain classes		0.006	3.872	0.278	15.764	0.072	0.021	9.274	9.782	0.020	2.617	0.050	7.875	27.195	0.283	20.737

## Conclusions

Taking all of the above into account the following conclusions were made:

1. Among the various metallurgical by-products containing germanium and indium known in the world, germanium-indium drosses are characterized by a very rich chemical composition in terms of the content of Ge and In, amounting to up to 10 and 6% wt%,, respectively. This is a global phenomenon.
2. The grain size and chemical composition of Ge-In-D is complex: a mixture of metallic and oxidized particles, as well as low-melting alloys.
3. The main ingredients of Ge-In-D are Sn in the amount of approx. 27–36 wt%, Pb in the amount of approx. 19–29 wt%,, Zn - approx. 8–11 wt%,. and Cu - approx. 8–22 wt%,. However, the uniqueness of Ge-In-D results from the presence of Ge and In in large amounts, approximately 7–10 and 2–8% wt%, respectively.
4. Important components of Ge-In-D are also As, in the amount of approx. 1–10 wt%, and Fe, in the amount of approx. 3–4 wt%. Depending on the grain class, the chemical composition is not uniform, and the highest concentrations of Ge and In were recorded for the smallest grains. These are spherical grains, consisting of a “shell” (outer part) and a “grain” (inner part), and Ge and In accumulate in the “shell”.
5. Despite the fact that the concentrations of Ge and In in the smallest fractions, i.e. 0.040/0.056 and 0.00/0.040 mm, can be as high as approx. 14–15 and 15–16 wt%, respectively, all fractions of Ge-In-D are rich enough in Ge and In and the entire material as such is suitable for chemical processing, without the need for mechanical enrichment.

## Data Availability

The datasets used and analyzed during the current study are available from the corresponding author on reasonable request.
